# P46 A mixed methods study exploring general dental practitioners’ views and experiences of antimicrobial use and stewardship in Ireland

**DOI:** 10.1093/jacamr/dlaf118.053

**Published:** 2025-07-14

**Authors:** A Fleming, C Napier, C Jennings, C Da Mata, C Ryan, M Shah, T M Barbosa

**Affiliations:** Pharmaceutical Care Research Group, School of Pharmacy, University College Cork, Cork, Ireland; Pharmacy Department, Mercy University Hospital, Cork, Ireland; Pharmaceutical Care Research Group, School of Pharmacy, University College Cork, Cork, Ireland; Pharmaceutical Care Research Group, School of Pharmacy, University College Cork, Cork, Ireland; Cork Dental School and Hospital, University College Cork, Cork, Ireland; Health Service Executive, Dublin, Ireland; Pharmaceutical Care Research Group, School of Pharmacy, University College Cork, Cork, Ireland; Health Service Executive, Cork, Ireland; Pharmaceutical Care Research Group, School of Pharmacy, University College Cork, Cork, Ireland

## Abstract

**Background:**

Antimicrobial prescribing in dentistry contributes to approximately 10% of overall antibiotic prescribing in primary care, which is significant. There is an opportunity to enhance antimicrobial stewardship (AMS) in dentistry practice in Ireland to address this. In order to understand the factors influencing antimicrobial prescribing by dentists, their views and experiences must be explored.

**Objectives:**

To conduct a mixed methods study to explore general dental practitioner’s views and experiences regarding antimicrobial use and antimicrobial resistance (AMR).

**Methods:**

An explanatory sequential mixed methods study was conducted. First, a survey exploring dental antimicrobial prescribing and views on antimicrobial prescribing and AMR was emailed to Irish Dental Association members in September 2024. The survey findings were analysed descriptively. The findings helped to refine the topic guide of the subsequent qualitative, semi-structured interviews conducted with general dental practitioners in November/December 2024. The verbatim interview transcripts were analysed by thematic analysis (Braun and Clarke) and then mapped to the Theoretical Domains Framework. Ethics approval was obtained, and all participants provided written informed consent.

**Results:**

A total of 79 survey responses (62% female) were obtained and 12 interviews (six female) were conducted. The survey found that 45 (57%) dentists referred to the national health service antibiotic prescribing guidelines. Many dentists felt antibiotics are overprescribed in dentistry (61/78, 78.2% agree/strongly agree) and 59 (74.7%) agree/strongly agree that patients often expect to be prescribed an antibiotic. The results found that 41% (32/78) of respondents reported they never calculate a weight-based antibiotic dose for a child. The main domains reported were knowledge, environmental context and resources, memory, attention and decision-making, beliefs about consequences, beliefs about capabilities, social influence and social/professional role. Dentists reported the pressure from patients to prescribe antibiotics and also the lack of time to review and intervene on patients with infection. ‘Just in case’ antibiotic prescribing was noted in the survey and interview findings. Dentists interviewed noted the challenge when making decisions for infections not responding to the initial course of antibiotics and communicating with patients where English is not their first language. Challenges in dental interventions, or antibiotic compliance, in children or those with special needs were also noted as impacting on decisions. Many highlighted the importance of continuing professional development (CPD) and audits to improve antimicrobial prescribing practices.

**Conclusions:**

This study identified important social and contextual factors in general dental practice which influence the prescribing of antimicrobials. To support the development of AMS in dental practice it is important to engage with dentists to ensure initiatives are tailored to their setting. CPD for dentists, patient education and surveillance of antibiotic prescribing in dental practice are recommended.

